# The moderating role of hippocampal volume in the association between emotional abuse and peer victimization in adolescents with major depressive disorder

**DOI:** 10.1007/s00787-025-02737-2

**Published:** 2025-05-14

**Authors:** Kyung Hwa Lee, Mijeong Park, Jiyoon Shin, Jung Lee, Jae Hyun Yoo, Jeeyoung Chun, Jae-Won Kim

**Affiliations:** 1 Division of Child and Adolescent Psychiatry, Department of Psychiatry, Seoul National University Hospital, Seoul, Republic of Korea; 2 Department of Psychiatry, SMG-SNU Boramae Medical Center, Seoul, Republic of Korea; 3 Integrative Care Hub, Seoul National University Children’s Hospital, Seoul, Republic of Korea; 4 Department of Psychiatry, Seoul ST. Mary’s Hospital, The Catholic University of Korea, Seoul, Republic of Korea; 5 Division of Child and Adolescent Psychiatry, Department of Psychiatry, Seoul National University Hospital, Seoul National University College of Medicine, 103 Daehak-Ro, Jongno-Gu, Seoul, 03080 Republic of Korea

**Keywords:** Early life adversity, Peer victimization, Hippocampal volume, Major depression disorder, Adolescence

## Abstract

Given its vicious cycle of victimization, early life adversity (ELA) in childhood may be associated with peer victimization during adolescence. Both ELA and peer victimization have been suggested to be major risk factors for depression. Volumetric alterations in the hippocampus implicated in stress sensitivity have been reported in individuals with ELA and peer victimization. This cross-sectional study examined the moderating role of hippocampal volume in the association between ELA and peer victimization in adolescents with major depressive disorder (MDD). The sample included 78 adolescents with MDD (age M (SD) = 14.92 (1.54) years, 56 females). The Early Trauma Inventory-Short Form and Peer Victimization Scale were used to assess participants’ ELA and peer victimization, respectively. High-resolution structural T1 images were obtained using a Siemens 3T magnetic resonance scanner. Whole hippocampal volumes were segmented and calculated using the FreeSurfer 6.0. Correlation and moderation analyses were also performed. Emotional abuse, a type of ELA, was significantly correlated with peer victimization after controlling for age and sex. The association between emotional abuse and peer victimization was moderated by bilateral hippocampal volume in adolescents with MDD after controlling for age, sex, and intracranial volume. Additionally, the association between emotional abuse and peer victimization was stronger when the bilateral hippocampal volumes were larger. Our findings partially supported the concept of a vicious cycle of victimization, which may be a critical aspect of depression in adolescents. Furthermore, the moderation results suggested that hippocampal volume plays an important role in the victimization cycle in adolescents with MDD.

**Supplementary Information:**

The online version contains supplementary material available at 10.1007/s00787-025-02737-2.

## Introduction

Early life adversity (ELA), which involves exposure to negative environments and stressful situations (e.g., physical abuse, emotional abuse, and traumatic events) in childhood, has been recognized as a major risk factor for adolescent depression [1]. Peer victimization is also closely associated with depression in adolescence [2, 3]. The idea of a vicious cycle of victimization suggests that individuals experiencing ELA are at high risk of revictimization [4, 5]. Previous studies have reported that those who experienced ELA are more likely to encounter victimization in other social contexts, such as peer relationships [6]. For example, various types of ELA (e.g., physical abuse and emotional abuse) were positively correlated with peer victimization [7, 8]. Importantly, both ELA and peer victimization are salient stressors experienced during childhood and adolescence and are prevalent in adolescents with depression [9]. For these reasons, examining the relationship between the two in this sample may be particularly important. Thus, in this study, we focused on examining the relationship between ELA and peer victimization, as two major stressors involved in the cycle of victimization.

As stressors, ELA and peer victimization are also known to influence neurodevelopment. The hippocampus has been implicated as a key region affected by ELA because it is involved in stress susceptibility, emotional memory, and learning through the regulation of the hypothalamic–pituitary–adrenal axis and interaction with the amygdala and prefrontal cortical regions [10]. Furthermore, hippocampal development may be dependent on age, as evidenced by its sensitivity to stress in early childhood (three to five years of age) and throughout adolescence, and is related to dramatic alterations in these periods [11]. Therefore, the hippocampus may play a crucial role in both ELA and peer victimization, which occur during sensitive periods for neurodevelopment, such as childhood and adolescence.

Studies have reported linear relationships of hippocampal volume with ELA and peer victimization in adolescence. For instance, adolescents who were physically abused showed smaller hippocampal volumes compared to those who were not [12]. Another study also demonstrated that greater ELA (e.g., the sum of scores from various ELA types, including physical and emotional abuse) was associated with smaller hippocampal volumes in adolescence [13]. In contrast, adolescents who reported higher levels of ELA (e.g., a total ELA score on ELA subtypes, including physical, emotional, and sexual abuse) were more likely to have enlarged hippocampal volumes [14]. One possible limitation of some previous studies is the use of ELA measures without considering the differential effects of ELA subtypes on brain alterations. Given that research on adults has provided evidence of ELA-specific effects on brain alterations [11], more research is necessary to better understand the effects of ELA subtypes on adolescents’ hippocampal volume. Additionally, few studies have examined the impact of peer victimization on hippocampal volume. Young individuals who experienced peer victimization showed larger hippocampal volumes than those who did not [15]. Another study found no significant effect of peer victimization on hippocampal volumetric alterations [16]. Thus, research on the relationships between ELA, peer victimization, and hippocampal volume remains limited, and previous findings are inconclusive.

Unlike in the aforementioned studies, more complex relationships may exist between ELA, peer victimization, and hippocampal volumetric alterations. For example, despite the cycle of victimization [5], not all adolescents who have ELA experience peer victimization. The neurobiological susceptibility model posits that neurobiological moderation contributes to individual differences in responding to early adversity and stress [17]. Therefore, neurobiological susceptibility may strengthen the cycle of victimization, such as the association between ELA and peer victimization. Considering the role of the hippocampus in stress sensitivity and emotional learning [10], the hippocampus as a core region of neurobiological susceptibility may play an important role in the association between ELA and peer victimization. Specifically, we focused on hippocampal volume because it is well known to be vulnerable to early adversity and stress [11]. Thus, interactions between ELA subtypes and hippocampal volume could contribute to peer victimization in adolescents with depression. Based on this possibility, we proposed a moderation model (Fig. 2a), in which the hippocampal volume may moderate the association between ELA and peer victimization in adolescents with depression.

In this study, we examined the relationship between ELA subtypes (e.g., physical and emotional abuse) and peer victimization in adolescents with major depressive disorder (MDD) to find evidence supporting the cycle of victimization. Based on previous studies, we explored the relationship between hippocampal volume, ELA, and peer victimization. As proposed in the moderation model, we investigated the moderating role of hippocampal volume in linking ELA and peer victimization in adolescents with MDD. First, consistent with the victimization cycle, we predicted that ELA would be positively correlated with peer victimization in adolescents with MDD. Furthermore, we expected different relationships between ELA subtypes (e.g., physical and emotional abuse) and peer victimization. Second, despite the mixed findings in previous studies with adolescents, we expected to observe inverse relationships between hippocampal volume, ELA, and peer victimization because neurotoxic effects (e.g., neuronal atrophy and cell death) of stress-related glucocorticoids on the hippocampus contribute to reduction in the hippocampal volume [18]. Finally, based on the possibility that the hippocampus plays a role as neurobiological susceptibility, we hypothesized that the hippocampal volume would moderate the relationship between ELA and peer victimization.

## Methods

### Participants

In this cross-sectional study, a total of 95 adolescents with MDD in the age group of 12–17 years were recruited from the Seoul National University Hospital in Korea. Two participants withdrew their consent after screening, and one dropped out before the magnetic resonance imaging (MRI) assessment. Furthermore, 14 adolescents were excluded owing to incidental findings (e.g., arachnoid cysts; *n* = 3) and artifacts such as motion and noise (*n* = 11). Our final sample comprised 78 adolescents with MDD (mean age [standard deviation] = 14.92 [1.54] years; 56 females). Adolescents with MDD were diagnosed based on the criteria in the fifth edition of the Diagnostic and Statistical Manual of Mental Disorders [19] using the Kiddie Schedule for Affective Disorders and Schizophrenia for School-Age Children-Present and Lifetime Version [20, 21]. They were medication free/naïve and approximately 77% of them was at their first episode of depression when they participated in this study. Thirty-three adolescents with MDD had a current diagnosis of one or more comorbid anxiety disorders, including generalized anxiety disorder (*n* = 22), social phobia (*n* = 12), panic disorder (*n* = 5), separation anxiety disorder (*n* = 3), agoraphobia (*n* = 2) and specific phobia (*n* = 1). Additional comorbidities included tic disorder (*n* = 3), oppositional defiant disorder (*n* = 2), conduct disorder (*n* = 2), obsessive-compulsive disorder (*n* = 1), posttraumatic stress disorder (*n* = 1) and bulimia nervosa (*n* = 1).

Adolescents with MDD were excluded if they had (a) an intelligence quotient lower than 70, (b) any neurological or chronic medical conditions, (c) a history of psychotic disorders, including schizophrenia or bipolar disorder, (d) a history of eating disorders, (e) any developmental disorders, such as autism, (f) a history of alcohol or other substance abuse within the past six months, and (g) first-degree relatives with a history of bipolar I disorder. This study was approved by the Institutional Review Board for Human Subjects at the Seoul National University Hospital. The parents provided informed consent, and the adolescents provided assent using forms approved by the Seoul National University Hospital Review Board. All methods were performed in accordance with the relevant guidelines and regulations.

### Measures of early life adversity, peer victimization, and depressive symptoms

We assessed ELA using the Early Trauma Inventory-Short Form (ETI-SF) completed by the primary caregivers [22]. It comprises 27 items measuring 4 categories of traumatic events: general trauma (11 items), physical abuse (5 items), emotional abuse (5 items), and sexual abuse (6 items). Each item was scored dichotomously (yes = 1, no = 0). The sum of the scores for each category was computed. A validated Korean version of the ETI-SF with good internal consistency (Cronbach’s *α* = 0.803) was used in this study [23]. Given the relatively low prevalence of sexual abuse reported in a previous study [23], we did not assess sexual abuse. Thus, we used the sum of the scores for general trauma, physical abuse, and emotional abuse.

Peer victimization was measured using the Peer Victimization Scale (PVS), which includes six items on being a victim of negative physical (e.g., being hit and pushed) and verbal (e.g., being teased and laughed at) behaviors [24]. Each item consists of two opposing statements (e.g., “Some kids are often teased by other kids, BUT other kids are not teased by other kids”). The participants were asked to choose which statement better represents them and to rate the statement as “really true for them” or “sort of true for them.” The responses are scored on a scale of 1 to 4 (two statements × two scales for each item), with higher scores indicating higher levels of peer victimization. The validated Korean version of the PVS [25] demonstrated strong internal consistency (Cronbach’s *α* = 0.770) and was used in this study.

Depressive symptoms were assessed using the Children’s Depression Rating Scale-Revised (CDRS-R) [26], which is widely used to assess depression severity in children and adolescents. It includes 17 symptom-related items that are derived from the adult Hamilton Depression Rating Scale and rated by clinical interviewers based on a summary of child and parent reports and behavioral observations during the interview. Excellent internal consistency of the Korean version of the CDRS-R [27] has been reported (Cronbach’s *α* = 0.91). This scale had good internal consistency in this sample (Cronbach’s *α* = 0.93).

### MRI acquisition and T1 image processing

High-resolution structural T1 images were acquired using a Siemens 3T MR scanner (Trio Tim; Siemens, Erlangen, Germany) with a 12-channel birdcage head coil. We used a T1-weighted 3D gradient echo pulse sequence with magnetization-prepared rapid gradient-echo sequencing (TR = 1,900 ms, TE = 3.13 ms, flip angle = 9°, slice thickness = 0.9 mm, matrix size = 256 × 224 × 176). We visually inspected T1 images and excluded the data with excessive head motion and incidental findings (e.g., arachnoid cysts). The structural T1 image was processed using the FreeSurfer 6.0 package (https://surfer.nmr.mgh.harvard.edu/). We used the default processing pipeline, called “recon-all” (https://surfer.nmr.mgh.harvard.edu/fswiki/recon-all/). This pipeline includes motion correction, intensity normalization, Talairach transformation, and skull stripping. Details of these processing steps have been described in a previous study [28]. During this process, the hippocampus was automatically segmented, and the hippocampal volume and intracranial volume (ICV) were calculated. We inspected the processed T1 images and found erroneous white matter segmentation near the parietal cortex. To correct this, we used the “control points” (https://surfer.nmr.mgh.harvard.edu/fswiki/FsTutorial/ControlPoints_freeview/) and reran part of the “recon-all.” Subsequently, we confirmed whether the white matter segmentation was corrected.

### Statistical analysis

Given that our sample included more females (approximately 72%) than males, we examined sex differences in clinical measures and hippocampal volumes using independent samples *t*-tests and analysis of covariance. Owing to the relatively wide age range, we conducted correlation analyses to examine whether age was correlated with clinical measures and hippocampal volume. We also performed correlation analyses to test whether ELA and peer victimization were associated with the severity of depressive symptoms. We controlled for ICV in any analysis of the hippocampal volume. To test our first hypothesis regarding the cycle of victimization, we investigated the relationships between ELA (i.e., general trauma, physical abuse, and emotional abuse) and peer victimization, controlling for age and sex. To test the second hypothesis, we examined the correlations between ELA, peer victimization, and hippocampal volume by conducting correlation analyses after controlling for age, sex, and ICV. The PROCESS macro [29] was used to examine whether hippocampal volume moderated the association between ELA and peer victimization (Fig. 2a) after controlling for age, sex, and ICV. The variables included in this model were mean-centered. The Johnson–Neyman technique implemented in PROCESS was used to investigate significant interactions between ELA and hippocampal volumes. For significant interactions, simple slopes were estimated at + 1SD (high) and − 1SD (low) from the mean. Correlation and moderation analyses were corrected for multiple tests using the Bonferroni correction. Thus, significant results were determined based on Bonferroni-corrected *p* values (0.05 divided by the number of tests). For example, correlation analyses testing the first and second hypotheses, Bonferroni-corrected *p* values were 0.017 (0.05/3 tests [3 subtypes of ELA x 1 peer victimization]) and 0.006 (0.05/8 tests [ left and right hippocampal volumes x 4 stressors including 3 ELA subtypes and peer victimization]), respectively. For moderation analyses testing the third hypothesis, Bonferroni-corrected *p* value was 0.008 (0.05/6 [left and right hippocampal volumes x 3 ELA subtypes]). All statistical analyses were performed using IBM SPSS version 25.0 (SPSS Inc., Chicago, IL, USA).

## Results

### Participant characteristics: demographic, clinical, and hippocampal volume information

Table 1 presents the participants’ demographic, clinical, and hippocampal volume characteristics. We did not find sex differences in any measures of ELA, peer victimization, depressive symptoms, or hippocampal volume on either side (all *ps* > 0.17). Age was correlated with depressive symptom severity (*r* = 0.25, *p* = 0.03) but not with ELA (ETI-SF subscales), peer victimization, or hippocampal volume (all *ps* > 0.08). Older adolescents with MDD were more depressed than younger adolescents with MDD. Depressive symptom severity assessed by the CDRS-R was significantly correlated with peer victimization (partial *r* = 0.41, *p* < 0.001) but not with any category of ELA (*ps* > 0.10) after controlling for age and sex.

**Table 1:** Demographic information, questionnaire scores, and hippocampal volume

Variable		Full sample (*N* = 78)
Demographic information		
Female, n (%)		56 (71.8)
Age, mean ± SD, median (IQR)		14.92 ± 1.54, 15.0 (2.0)
Depressive symptoms		
CDRS-R, mean ± SD, median (IQR)		58.77 ± 11.69, 59.0 (14.0)
Early life adversity		
ETI-SF general trauma, mean ± SD, median (IQR)		1.13 ± 1.26, 1.0 (2.0)
ETI-SF physical abuse, mean ± SD, median (IQR)		1.71 ± 1.42, 2.0 (3.0)
ETI-SF emotional abuse, mean ± SD, median (IQR)		1.90 ± 1.73, 1.5 (3.0)
Peer victimization		
PVS, mean ± SD, median (IQR)		2.21 ± 0.59, 2.17 (0.84)
Hippocampal volume (mm^3^)		
Right hippocampus, mean ± SD, median (IQR)		4336.58 ± 348.31, 3920.10 (457.82)
Left hippocampus, mean ± SD, median (IQR)		3929.64 ± 429.04, 4294.70 (542.78)
ICV, mean ± SD, median (IQR)		1532970.25 ± 140466.97, 1512534.26 (219921.53)

Note. SD, Standard Deviation; IQR, Interquartile Range; CDRS-R, Children’s Depression Rating Scale-Revised; ETI-SF, Early Trauma Inventory-Short Form; PVS, Peer-Victimization Scale; ICV, Intracranial Volume

### Correlations between early life adversity subtypes and peer victimization

**Fig. 1:**
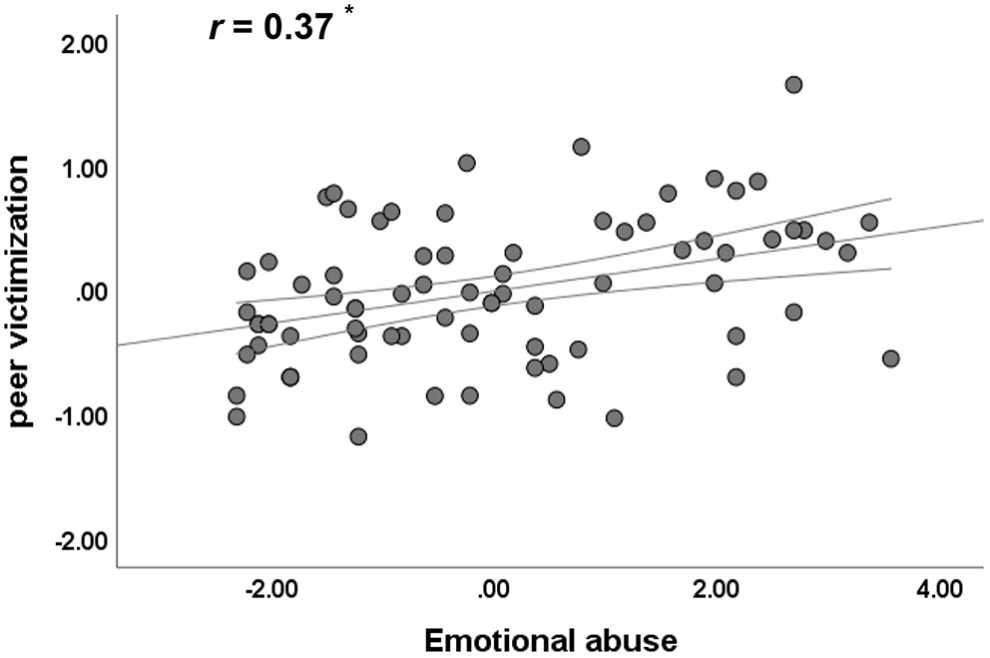
Partial correlation between emotional abuse and peer victimization in adolescents with MDD, controlling for age and sex. ^*^ indicates significant at Bonferroni-corrected *p* < 0.017

Emotional abuse was significantly correlated with peer victimization (partial *r* = 0.37, uncorrected *p* = 0.001, Bonferroni-corrected *p* < 0.017) (Fig. 1), whereas physical abuse and general trauma were not correlated with peer victimization (partial *r* = 0.10, *p* = 0.41, and partial *r* = 0.16, *p* = 0.17, respectively) after controlling for age and sex (for more details, see Supplementary Table S1).

### Correlations of hippocampal volumes with early life adversity subtypes and peer victimization

We performed correlation analyses to test our second hypothesis. Hippocampal volumes were not significantly correlated with any type of ELA (all *ps* > 0.12) or with peer victimization (all *ps* > 0.93) after controlling for age, sex, and ICV (see Supplementary Table S2).

### Moderation results: relationship between emotional abuse and peer victimization moderated by hippocampal volume

**Fig. 2:**
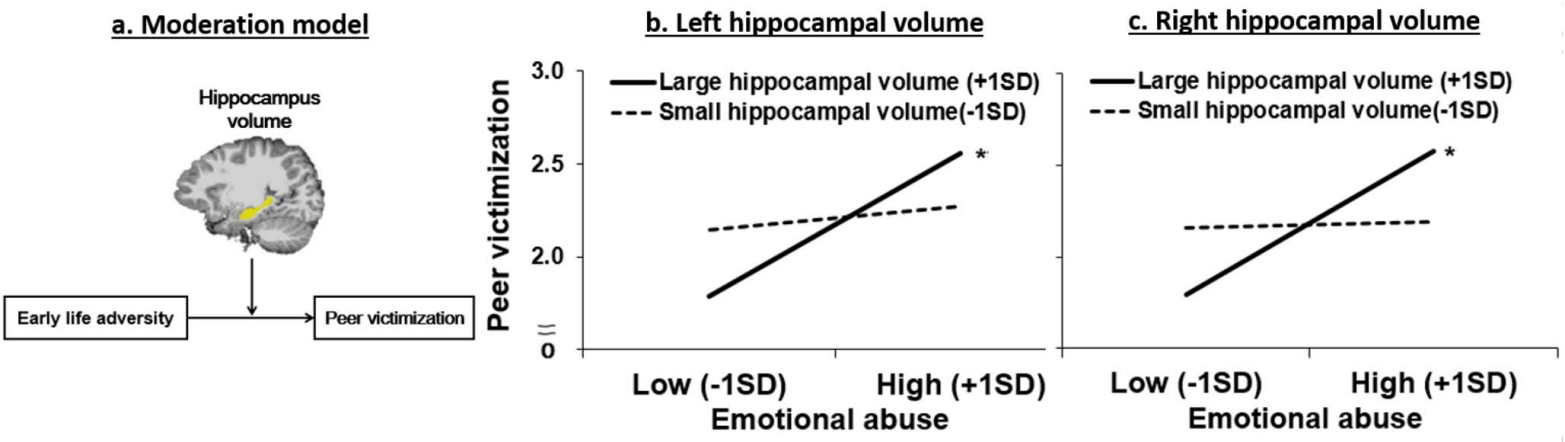
**a**. The proposed moderation model describing the possible associations between early life adversity, peer victimization and hippocampal volumes. **b**. The result of the moderation model: Emotional abuse x left hippocampal volume predicting peer victimization, controlling for age, sex, and ICV. **c**. The result of the moderation model: Emotional abuse x right hippocampal volume predicting peer victimization, controlling for age, sex, and ICV. Note. ICV, Intracranial Volume, +1SD, mean + 1SD; -1SD, mean-1SD ^*^ indicates significant at Bonferroni-corrected *p* < 0.008

Given that emotional abuse was the only type of ELA that was significantly correlated with peer victimization, we examined the moderating role of hippocampal volume in the association between emotional abuse and peer victimization. As shown in Table 2a, the left hippocampal volume significantly moderated the relationship between emotional abuse and peer victimization after controlling for age, sex, and ICV. A simple slope analysis using the Johnson–Neyman method showed that the association between emotional abuse and peer victimization was stronger when the left hippocampal volume was larger (mean + 1SD; *b* = 0.22, standard error [*SE*] = 0.05, uncorrected *p* < 0.001, Bonferroni-corrected *p* < 0.008) than when it was smaller (mean–1SD; *b* = 0.04, *SE* = 0.05, *p* = 0.41). Figure 2b illustrates the interaction effect by depicting two simple slopes.

**Table 2:** The results of the moderation analysis, controlling for age, sex, and ICV

a. Emotional abuse x left hippocampus volume predicting peer victimization					
	△R^2^	b	SE	*t*	*p*
Main effects	0.19				
Emotional abuse		0.13	0.03	3.74	0.0004^*^
Left hippocampus volume		-0.05	0.21	-0.25	0.80
Age		0.05	0.04	1.23	0.22
Sex		-0.05	0.15	-0.33	0.74
ICV		-0.07	0.06	-1.11	0.27
Interaction effect	0.09				
** Emotional abuse x L hipp volume**		0.26	0.09	3.00	**0.004** ^*^
Model R^2^ = 0.28, F(6, 71) = 4.67, *p* < 0.001^*^					
**b. Emotional abuse x right hippocampus volume predicting peer victimization**					
	△**R**^2^	**b**	**SE**	***t***	***p***
Main effect	0.19				
Emotional abuse		0.12	0.03	3.42	0.001^*^
Right hippocampus volume		0.01	0.18	0.06	0.95
Age		0.05	0.04	1.23	0.22
Sex		-0.11	0.15	-0.72	0.47
ICV		-0.09	0.06	-1.48	0.14
Interaction effect	0.12				
**Emotional abuse x R hipp volume**		0.26	0.07	3.55	**0.0007** ^*^
Model R^2^ = 0.31, F(6, 71) = 5.43, *p* < 0.001^*^					

Note. ICV, Intracranial Volume; L, left; R, right; Hipp, Hippocampus; SE, Standard Error

^*^ indicates significant at Bonferroni-corrected *p* < 0.008

The right hippocampal volume also showed a similar pattern, displaying a significant moderating effect on the association between emotional abuse and peer victimization after controlling for age, sex, and ICV (Table 2b). The simple slope analysis revealed that the association between emotional abuse and peer victimization was stronger when the right hippocampal volume was larger (mean + 1SD; *b* = 0.23, *SE* = 0.04, uncorrected *p* < 0.001, Bonferroni-corrected *p* < 0.008) than when it was smaller (mean–1SD; *b* = 0.01, *SE* = 0.05, *p* = 0.87). Figure 2c illustrates the interaction effect by depicting two simple slopes.

Despite insignificant correlations between general trauma, physical abuse, and peer victimization, we investigated whether hippocampal volume moderated the associations between general trauma and peer victimization, and between physical abuse and peer victimization. There were no significant moderating effects of hippocampal volumes on the associations between general trauma and peer victimization, and between physical abuse and peer victimization. These results are presented in Supplementary Table S3 and Table S4.

### Additional analyses

#### Correlation analyses, controlling for depressive symptoms and physical abuse

The CDRS-R scores were entered as covariates to control for depressive symptom severity. The correlation between emotional abuse and peer victimization remained significant after controlling for age, sex, and depressive symptom severity (partial *r* = 0.33, *p* = 0.004). Given the significant correlation between emotional and physical abuse (partial *r* = 0.41, *p* < 0.001), we examined the unique relationship between emotional abuse and peer victimization by adjusting for physical abuse. Emotional abuse was still significantly correlated with peer victimization after controlling for age, sex, and physical abuse (partial *r* = 0.39, *p* < 0.001). These correlation results are presented in Supplementary Table S1.

#### Moderation analyses, controlling for depressive symptom severity

Given the significant correlation between depressive symptoms and peer victimization, we conducted moderation analyses to control for the potential effects of depressive symptom severity. The moderation results described above remained significant after controlling for depressive symptom severity, indicating that it did not affect the findings (see Supplementary Table S5).

#### Moderation analyses, controlling for physical abuse

We conducted additional moderation analyses to control for the effects of physical abuse. The results remained significant after controlling for physical abuse, indicating that it did not influence the moderation results (see Supplementary Table S6).

## Discussion

We examined the relationship between ELA, peer victimization, and hippocampal volume. Furthermore, we investigated whether the hippocampal volume, known to be involved in stress susceptibility and emotional learning, moderated the relationship between ELA and peer victimization in adolescents with MDD, who were more likely to have ELA and peer victimization. Only emotional abuse, a type of ELA, was significantly correlated with peer victimization. In support of the moderation model we proposed, the association between emotional abuse and peer victimization was moderated by hippocampal volume in adolescents with MDD.

Consistent with the vicious cycle of victimization [5], emotional abuse was positively correlated with peer victimization. This result aligns with previous studies finding that emotional abuse was significantly associated with peer victimization [8, 30, 31]. There were several reasons for this finding. First, emotional abuse typically involves humiliation, denigration, and isolation [32], which may hurt individuals’ sense of self-worth and self-image [33]. Second, emotional abuse tends to be sustained and involves maladaptive interpersonal interaction patterns, such as inappropriate and inconsistent caregiver–child interactions. For these reasons, adolescents who were emotionally abused may develop maladaptive cognitive processes, such as negative self-concept, negative attributional styles, and interpersonal difficulties [34, 35]. These characteristics may increase the probability of being victimized by peers. As depression is characterized by interpersonal or social problems and negative cognitive styles, the association between emotional abuse and peer victimization may be particularly salient in adolescents with MDD.

Surprisingly, physical abuse and general trauma were not significantly associated with peer victimization among adolescents with MDD. Given that our sample comprised adolescents with MDD, who are known to be more likely to experience peer victimization than to perpetrate bullying [36], the association between physical abuse and peer victimization may not have been robust enough to be identified in this study. However, because of our moderate sample size, this interpretation should be accepted with caution. General trauma in the ETI-SF includes several traumatic events, such as natural disasters, serious accidents, and parental divorce. This may be one reason for the lack of significant findings regarding the relationship between general trauma and peer victimization among adolescents with MDD. Further research with a larger sample size is needed to examine the relationships between physical abuse, traumatic events, and peer victimization.

As hypothesized, hippocampal volume moderated the relationship between emotional abuse and peer victimization. This result supports the idea that neurobiological markers may moderate the relationship between ELA and peer victimization [6]. More specifically, emotional abuse was more positively associated with peer victimization when hippocampal volumes were larger. Given that larger hippocampal volumes are associated with greater sensitivity to both negative and positive social contexts [37, 38], they may reflect greater sensitivity to social stressors. Thus, adolescents who experience emotional abuse and have larger hippocampal volumes may be more sensitive to social stressors and more likely to experience peer victimization. This finding underscores the role of the hippocampus as a key region of neurobiological susceptibility [37] in the associations among different social stressors, such as emotional abuse and peer victimization, in adolescents with MDD.

Inconsistent with some previous studies [12–14] and our hypothesis, hippocampal volumes were not significantly correlated with the ELA subtypes (e.g., physical and emotional abuse) or peer victimization in this study. This may be attributed to the sample characteristics. We only included adolescents with MDD, who may have had relatively small variations in ELA and peer victimization scores and hippocampal volumes. Another possible reason could be related to age-dependent hippocampal development. As the hippocampus is still developing during adolescence [39], the scarring effects of ELA and peer victimization on the hippocampal structure can be delayed until emerging adulthood. Therefore, the direct relationship of the hippocampal volume with ELA and peer victimization may not manifest in adolescence. Further research using samples with more variations in the study variables may be needed to examine the linear relationships between these variables.

This study has some limitations. First, because our sample included only adolescents with MDD, the generalization of our findings (i.e., the moderating role of hippocampal volume in the relationship between emotional abuse and peer victimization) to community or clinical samples with different psychological disorders may be limited. Second, we assessed ELA and peer victimization using retrospective questionnaires, which might have generated recall bias. Third, the measures collected limited information by focusing on whether the participants experienced ELA and peer victimization. However, we did not address the time of occurrence, frequency, and severity of ELA and peer victimization, which may be important factors related to hippocampal development. Fourth, given that ELA was reported by caregivers who might be perpetrators and not disclose their past abuse, this may generate social desirability bias, which is a tendency to underreport unsocially desirable responses and to give more socially desirable responses. Child-reported or third parties-reported ELA were not collected in this study, so we were unable to test validity and reliability of ELA rated by different informants. Future research may be necessary for investigating whether our main findings are replicated by ELA collected from different informants. Fifth, we did not gather data on other socioenvironmental factors, such as neighborhood and deprivation, which may have influenced hippocampal development. Sixth, although the hippocampus is connected and interacts with other brain regions, including the amygdala and prefrontal cortical regions, to regulate stress responses, we only examined the hippocampal volume. This limits our understanding of the interactive role of the hippocampus with the amygdala and prefrontal cortices in response to stressors, such as emotional abuse and peer victimization. Finally, other factors, such as a history of medication use and comorbidity with anxiety or posttraumatic stress disorders, may affect changes in hippocampal volume. However, limited information about medication history and relatively small sample size restricted us from exploring whether these factors influence hippocampal volumetric changes in this study.

Despite these limitations, this study is one of the first to examine the cycle of victimization in adolescents with MDD and the moderating role of neurobiological markers such as hippocampal volume in the relationship between ELA and peer victimization. Our findings provide supportive evidence for the cycle of victimization by demonstrating a significant correlation between emotional abuse and peer victimization. We also report novel findings regarding the role of the hippocampus in neurobiological susceptibility regarding the cycle of victimization in adolescents with depression. Our findings have several important clinical implications. Given the significant correlation between emotional abuse and peer victimization, it is important to help adolescents who experience emotional abuse develop adaptive interpersonal skills and positive cognitive styles, thereby contributing to the prevention of subsequent victimization.

## Electronic supplementary material

Below is the link to the electronic supplementary material.

Supplementary Material 1

## Data Availability

The data that support the findings of this study are available from the corresponding author upon reasonable request.
