# Supplementary material for: The moderating role of hippocampal volume in the association between emotional abuse and peer victimization in adolescents with major depressive disorder
Source: Eur Child Adolesc Psychiatry. 2025 May 14;34(10):3175–83. doi: 10.1007/s00787-025-02737-2 (PMC12592281; doi:10.1007/s00787-025-02737-2)
Supplement: Supplementary file 1 — Supplementary Material 1 [file 787_2025_2737_MOESM1_ESM.docx]

**Supplementary materials**

**The moderating role of hippocampal volume in the association between emotional abuse and peer victimization**

**in adolescents with major depressive disorder**

Kyung Hwa Lee, Mijeong Park, Jiyoon Shin, Jung Lee, Jae Hyun Yoo,

Jeeyoung Chun, Jae-Won Kim

**Supplementary tables**

**Table S1. Correlations between early life adversity subtypes and peer victimization**

| **a. Zero-order correlations** |  |  |  |  |  |
| --- | --- | --- | --- | --- | --- |
|  | 1 | 2 | 3 | 4 | 5 |
| 1. ETI-SF_general trauma | - |  |  |  |  |
| 2. ETI-SF_physical abuse | 0.21 | - |  |  |  |
| 3. ETI-SF_emotional abuse | 0.1 | 0.40^a^ | - |  |  |
| 4. PVS_peer victimization | 0.12 | 0.07 | **0.41^*^** | - |  |
| 5. CDRS-R_depressive symptom | 0.06 | 0.04 | 0.22 | 0.46 ^a^ | - |
|  |  |  |  |  |  |
| **b. Partial correlations, controlling for age and sex** | | |  |  |  |
|  | 1 | 2 | 3 | 4 | 5 |
| 1. ETI_general trauma | - |  |  |  |  |
| 2. ETI_physical abuse | 0.22 | - |  |  |  |
| 3. ETI_emotional abuse | 0.14 | 0.41^a^ | - |  |  |
| 4. PVS_peer victimization | 0.16 | 0.1 | **0.37^*^** | - |  |
| 5. CDRS-R_depressive symptom | 0.17 | 0.05 | 0.19 | 0.41^a^ | - |
|  |  |  |  |  |  |
| **c. Partial correlations, controlling for age, sex, and depressive symptom severity** | | | | | |
|  | 1 | 2 | 3 | 4 |  |
| 1. ETI_general trauma | - |  |  |  |  |
| 2. ETI_physical abuse | 0.21 | - |  |  |  |
| 3. ETI_emotional abuse | 0.11 | 0.41 ^a^ | - |  |  |
| 4. PVS_peer victimization | 0.1 | 0.1 | **0.33^*^** | - |  |

^*^ indicates significant at Bonferroni-corrected *p* < 0.017

^a^ indicates significant at Bonferroni-corrected *p* < 0.006

Note. ETI-SF, Early Trauma Inventory-Short Form; PVS, Peer-Victimization Scale; CDRS-R, Children’s Depression Rating Scale-Revised

**Table S2. Correlations between hippocampal volume, early life adversity subtypes, and peer victimization**

| **a. Partial correlations between hippocampal volume, early life adversity, and peer victimization, controlling for ICV** | | | | | | | |
| --- | --- | --- | --- | --- | --- | --- | --- |
|  | 1 | 2 | 3 | 4 | 5 | 6 | 7 |
| 1. Left hippocampus volume | - |  |  |  |  |  |  |
| 2. Right hippocampus volume | **0.71^*^** | - |  |  |  |  |  |
| 3. ETI_general trauma | -0.14 | -0.06 | - |  |  |  |  |
| 4. ETI_physical abuse | 0.08 | 0.18 | 0.22 | - |  |  |  |
| 5. ETI_emotional abuse | 0.05 | 0.08 | 0.1 | 0.39 | - |  |  |
| 6. PVS_peer victimization | 0.02 | 0.02 | 0.12 | 0.07 | 0.41 | - |  |
| 7. CDRS-R_depressive symptom | -0.01 | 0.004 | 0.06 | 0.07 | 0.25 | 0.47 | - |
|  |  |  |  |  |  |  |  |
| **b. Partial correlations between hippocampal volume, early life adversity, and peer victimization, controlling for age, sex, and ICV** | | | | | | | |
|  | 1 | 2 | 3 | 4 | 5 | 6 | 7 |
| 1. Left hippocampus volume | - |  |  |  |  |  |  |
| 2. Right hippocampus volume | **0.71^*^** | - |  |  |  |  |  |
| 3. ETI_general trauma | -0.12 | -0.04 | - |  |  |  |  |
| 4. ETI_physical abuse | 0.09 | 0.18 | 0.21 | - |  |  |  |
| 5. ETI_emotional abuse | 0.02 | 0.06 | 0.13 | 0.40 | - |  |  |
| 6. PVS_peer victimization | -0.01 | -0.01 | 0.15 | 0.07 | 0.39 | - |  |
| 7. CDRS-R_depressive symptom | -0.05 | -0.03 | 0.09 | 0.08 | 0.21 | 0.44 | - |

^*^ indicates significant at Bonferroni-corrected *p* < 0.006

Note. ICV, Intracranial Volume; ETI-SF, Early Trauma Inventory-Short Form; PVS, Peer-Victimization Scale; CDRS-R, Children’s Depression Rating Scale-Revised

**Table S3**. The results of the moderation analysis, controlling for age, sex, and ICV

| **a. General trauma x left hippocampus volume predicting peer victimization** | | | | | |
| --- | --- | --- | --- | --- | --- |
|  | △R^2^ | b | SE | *t* | *p* |
| Main effects | 0.07 |  |  |  |  |
| General trauma |  | 0.09 | 0.05 | 1.60 | 0.11 |
| Left hippocampus volume |  | -0.18 | 0.25 | -0.71 | 0.48 |
| Age |  | 0.08 | 0.04 | 1.85 | 0.07 |
| Sex |  | -0.15 | 0.17 | -0.90 | 0.37 |
| ICV |  | -0.04 | 0.06 | -0.59 | 0.56 |
| Interaction effect | 0.05 |  |  |  |  |
| General trauma x L hipp volume |  | 0.25 | 0.12 | 2.10 | 0.04^a^ |
| Model R^2^ = 0.12, F(6, 71) = 1.63, p = 0.15 | | | | |  |
| **b. General trauma x right hippocampus volume predicting peer victimization** | | | | | |
|  | △R^2^ | b | SE | *t* | *p* |
| Main effects | 0.07 |  |  |  |  |
| General trauma |  | 0.08 | 0.05 | 1.46 | 0.15 |
| Right hippocampus volume |  | -0.09 | 0.20 | -0.43 | 0.67 |
| Age |  | 0.08 | 0.04 | 1.83 | 0.07 |
| Sex |  | -0.16 | 0.17 | -0.92 | 0.36 |
| ICV |  | -0.04 | 0.06 | -0.60 | 0.55 |
| Interaction effect | 0.06 |  |  |  |  |
| General trauma x R hipp volume |  | 0.22 | 0.10 | 2.22 | 0.03^a^ |
| Model R^2^ = 0.13, F(6, 71) = 1.73, p = 0.13 | | | | |  |

^a^ did not survive after the Bonferroni correction

Note. ICV, Intracranial Volume; L, left; R, right; Hipp, Hippocampus; SE, Standard Error

**Table S4**. The results of the moderation analysis, controlling for age, sex, and ICV

| **a. Physical abuse x left hippocampus volume predicting peer victimization** | | | | | |
| --- | --- | --- | --- | --- | --- |
|  | △R^2^ | b | SE | *t* | *p* |
| Main effects | 0.05 |  |  |  |  |
| Physical abuse |  | 0.03 | 0.05 | 0.61 | 0.54 |
| Left hippocampus volume |  | -0.08 | 0.24 | -0.33 | 0.74 |
| Age |  | 0.07 | 0.04 | 1.61 | 0.11 |
| Sex |  | -0.12 | 0.17 | -0.71 | 0.48 |
| ICV |  | -0.03 | 0.07 | -0.50 | 0.62 |
| Interaction effect | 0.03 |  |  |  |  |
| Physical abuse x L hipp volume |  | 0.21 | 0.14 | 1.51 | 0.14 |
| Model R^2^ = 0.11, F(6, 71) = 1.05, p = 0.40 | | | | |  |
| **b. Physical abuse x right hippocampus volume predicting peer victimization** | | | | | |
|  | △R^2^ | b | SE | *t* | *p* |
| Main effects | 0.05 |  |  |  |  |
| Physical abuse |  | 0.02 | 0.05 | 0.35 | 0.72 |
| Right hippocampus volume |  | 0.04 | 0.21 | 0.21 | 0.84 |
| Age |  | 0.07 | 0.04 | 1.51 | 0.13 |
| Sex |  | -0.17 | 0.17 | -0.98 | 0.33 |
| ICV |  | -0.06 | 0.07 | -0.91 | 0.37 |
| Interaction effect | 0.05 |  |  |  |  |
| Physical abuse x R hipp volume |  | 0.25 | 0.13 | 1.94 | 0.06 |
| Model R^2^ = 0.10, F(6, 71) = 1.31, p = 0.26 | | | | |  |

Note. ICV, Intracranial Volume; L, left; R, right; Hipp, Hippocampus; SE, Standard Error

**Table S5**. The results of the moderation analysis, controlling for age, sex, ICV, and depressive symptom severity

| **a. Emotional abuse x left hippocampus volume predicting peer victimization** | | | | | |
| --- | --- | --- | --- | --- | --- |
|  | △R^2^ | b | SE | *t* | *p* |
| Main effects | 0.32 |  |  |  |  |
| Emotional abuse |  | 0.11 | 0.03 | 3.20 | 0.002 |
| Left hippocampus volume |  | -0.01 | 0.20 | -0.06 | 0.95 |
| Age |  | 0.02 | 0.04 | 0.51 | 0.61 |
| Sex |  | -0.04 | 0.15 | -0.29 | 0.78 |
| ICV |  | -0.03 | 0.06 | -0.58 | 0.57 |
| Depressive symptom severity |  | 0.02 | 0.005 | 3.08 | 0.003 |
| Interaction effect | 0.05 |  |  |  |  |
| Emotional abuse x L hipp volume |  | 0.21 | 0.09 | 2.43 | 0.018 |
| Model R^2^ = 0.37, F(7, 70) = 5.83, p < 0.0001 | | | | |  |
| **b. Emotional abuse x right hippocampus volume predicting peer victimization** | | | | | |
|  | △R^2^ | b | SE | *t* | *p* |
| Main effects | 0.32 |  |  |  |  |
| Emotional abuse |  | 0.10 | 0.03 | 2.98 | 0.004 |
| Right hippocampus volume |  | 0.02 | 0.17 | 0.11 | 0.91 |
| Age |  | 0.02 | 0.04 | 0.56 | 0.58 |
| Sex |  | -0.09 | 0.14 | -0.61 | 0.55 |
| ICV |  | -0.05 | 0.06 | -0.87 | 0.39 |
| Depressive symptom severity |  | 0.02 | 0.005 | 2.86 | 0.006 |
| Interaction effect | 0.07 |  |  |  |  |
| Emotional abuse x R hipp volume |  | 0.20 | 0.07 | 2.85 | 0.006 |
| Model R^2^ = 0.39, F(7, 70) = 6.30, p < 0.0001 | | | | |  |

Note. ICV, Intracranial Volume; L, left; R, right; Hipp, Hippocampus; SE, Standard Error

**Table S6**. The results of the moderation analysis, controlling for age, sex, ICV, and physical abuse

| **a. Emotional abuse x left hippocampus volume predicting peer victimization** | | | | | |
| --- | --- | --- | --- | --- | --- |
|  | △R^2^ | b | SE | *t* | *p* |
| Main effects | 0.20 |  |  |  |  |
| Emotional abuse |  | 0.15 | 0.04 | 4.00 | 0.0002 |
| Left hippocampus volume |  | -0.03 | 0.21 | -0.13 | 0.89 |
| Age |  | 0.04 | 0.04 | 1.04 | 0.30 |
| Sex |  | -0.06 | 0.15 | -0.40 | 0.69 |
| ICV |  | -0.06 | 0.06 | -1.10 | 0.28 |
| Physical abuse |  | -0.06 | 0.05 | -1.37 | 0.17 |
| Interaction effect | 0.10 |  |  |  |  |
| Emotional abuse x L hipp volume |  | 0.28 | 0.09 | 3.20 | 0.002 |
| Model R^2^ = 0.30, F(7, 70) = 4.32, p < 0.001 | | | | |  |
| **b. Emotional abuse x right hippocampus volume predicting peer victimization** | | | | | |
|  | △R^2^ | b | SE | *t* | *p* |
| Main effects | 0.20 |  |  |  |  |
| Emotional abuse |  | 0.14 | 0.04 | 3.76 | 0.003 |
| Right hippocampus volume |  | 0.06 | 0.18 | 0.35 | 0.73 |
| Age |  | 0.04 | 0.04 | 1.01 | 0.31 |
| Sex |  | -0.12 | 0.15 | -0.79 | 0.43 |
| ICV |  | -0.09 | 0.06 | -1.56 | 0.12 |
| Physical abuse |  | -0.07 | 0.05 | -1.51 | 0.14 |
| Interaction effect | 0.14 |  |  |  |  |
| Emotional abuse x R hipp volume |  | 0.28 | 0.07 | 3.79 | 0.0003 |
| Model R^2^ = 0.34, F(7, 70) = 5.07, p < 0.001 | | | | |  |

Note. ICV, Intracranial Volume; L, left; R, right; Hipp, Hippocampus; SE, Standard Error
